# Low Bilirubin Levels Indicate a High Risk of Cerebral Deep White Matter Lesions in Apparently Healthy Subjects

**DOI:** 10.1038/s41598-018-24917-8

**Published:** 2018-04-24

**Authors:** Satoshi Higuchi, Yusuke Kabeya, Junko Uchida, Kiyoe Kato, Nobuhiro Tsukada

**Affiliations:** 1 Department of Cardiology, Kyorin University Hospital, Tokyo, Japan; 2 Division of General Internal Medicine, Department of Internal Medicine, Tokai University, Kanagawa, Japan; 3 Department of Home Care Medicine, Saiyu Clinic, Saitama, Japan; 4 Department of Internal Medicine, Tokyo Saiseikai Central Hospital, Tokyo, Japan

## Abstract

Recent studies have reported that deep white matter lesions (DWMLs) on magnetic resonance imaging scans are related to the risk of developing impaired cognitive function in future. Bilirubin exhibits a potent antioxidant effect and an inverse relationship has been reported between bilirubin levels and the risk of several atherosclerotic diseases; however, there is limited evidence with regard to the effect of bilirubin levels on cerebrovascular diseases including DWMLs. This cross-sectional study included 1121 apparently healthy Japanese adults. The subjects were divided into three groups according to their bilirubin levels (low, <0.5 mg/dl; intermediate, ≥0.5 mg/dl and <1.0 mg/dl; and high, ≥1.0 mg/dl). The severity of DWMLs was evaluated according to Fazekas scale and their relation to bilirubin levels was examined. The association between bilirubin levels and the presence of severe DWMLs was assessed using multivariate logistic regression analysis. The analysis revealed that the low- and intermediate bilirubin groups indicated 2.36- and 1.33-fold increase in the prevalence of severe DWMLs compared with the high-bilirubin group, respectively (95% confidence interval (CI): 1.12–4.97 (the low-bilirubin group), 95% CI: 0.85–2.07 (the intermediate-bilirubin group). In conclusion, low total bilirubin levels could be associated with a high prevalence of severe DWMLs in apparent healthy subjects.

## Introduction

An ischemic change in the deep white matter is often documented even in apparently healthy subjects. The deep white matter lesions (DWMLs) are reported to be related to the risk of developing impaired cognitive function and stroke^1–3^. The number of elderly and super-elderly subjects has increased worldwide, particularly in developed countries. Early documentation and intervention may be required in case of such lesions if the subjects are presented with treatable atherosclerotic risk factors. We have formerly reported the strong relationship between DWMLs and visceral-to-subcutaneous fat ratio, which is determined by abdominal computed tomography^4^. Although the ratio is useful for the risk stratification of cerebral ischemic change, the cost and the exposure to radiation are challenging. Recent studies have indicated a possible relationship between low bilirubin levels and cardiovascular diseases^5,6^. Similar findings have been reported in the relationship of bilirubin levels with stroke although it remains controversial whether the relationship is causal^7,8^. In general, bilirubin is an end product of heme metabolism and can be a toxic waste product in subjects with liver impairment such as liver cirrhosis. However, previous studies revealed that bilirubin exhibited potent antioxidant and anti-inflammatory effects^9,10^. The measurement of serum bilirubin levels is easy, inexpensive, and less invasive. If the relationship between bilirubin levels and DWMLs is confirmed, an early documentation of subjects at a high risk of dementia may be possible. The present study was conducted to examine the effectiveness of bilirubin as a marker for DWHLs.

## Results

### The Subjects’ Characteristics

The present study included 1121 subjects (age, 62 ± 10 years; male, 67%). The background characteristics in terms of the total bilirubin levels are shown in Table 1. There was no significant difference among the groups in terms of atherosclerotic risk factors.

**Table 1:** The Subjects’ Characteristics.

	All	Total Bilirubin			*p* value
	(n = 1121)	Low (n = 83)	Intermediate (n = 818)	High (n = 220)	All
Age, year	62 ± 10	59 ± 11	63 ± 10	61 ± 9	0.001
Male, n (%)	756 (67)	50 (60)	535 (65)	171 (78)	0.001
Height, cm	164 ± 8	164 ± 9	164 ± 9	166 ± 8	0.001
Weight, kg	63 ± 12	65 ± 13	62 ± 12	64 ± 11	0.008
Body Mass Index, kg/m^2^	23 ± 3	24 ± 4	23 ± 3	23 ± 3	0.002
Systolic Blood Pressure (mmHg)	122 ± 19	120 ± 21	122 ± 19	123 ± 19	0.663
Diastolic Blood Pressure (mmHg)	75 ± 12	74 ± 11	75 ± 12	77 ± 12	0.112
Hypertension, n (%)	242 (22)	19 (23)	178 (22)	45 (20)	0.876
Diabetes Mellitus, n (%)	170 (15)	14 (17)	127 (16)	29 (13)	0.625
Dyslipidemia, n (%)	77 (7)	8 (10)	52 (6)	17 (8)	0.454
Hyperuricemia, n (%)	68 (6)	5 (6)	48 (6)	15 (7)	0.872
Never Smoker, n (%)	675 (60)	45 (54)	489 (60)	141 (64)	0.260
Past Smoker, n (%)	306 (27)	21 (25)	232 (28)	53 (24)	0.412
Current Smoker, n (%)	140 (12)	17 (20)	97 (12)	26 (12)	0.073
Alcohol Consumption					
None, n(%)	244 (22)	23 (28)	181 (22)	40 (18)	0.179
1–4 days a week, n (%)	522 (47)	44 (53)	379 (46)	99 (45)	0.445
5 days or more, n (%)	355 (32)	16 (19)	258 (32)	81 (37)	0.014
White Blood Cell, /mm^3^	5500 ± 1600	5900 ± 1900	5400 ± 1600	5300 ± 1300	0.007
Hemoglobin, g/dl	14.0 ± 1.2	13.4 ± 1.4	13.9 ± 1.2	14.4 ± 1.2	<0.001
Platelet, x10^3^/mm^3^	220 ± 54	330 ± 58	221 ± 54	213 ± 49	0.030
Albumin, g/dl	4.6 ± 0.3	4.5 ± 0.2	4.6 ± 0.2	4.7 ± 0.3	<0.001
Uric Acid, mg/dl	5.6 ± 1.3	5.5 ± 1.3	5.6 ± 1.3	5.7 ± 1.3	0.231
Creatinine, mg/dl	0.8 ± 0.2	0.9 ± 0.4	0.8 ± 0.2	0.9 ± 0.2	0.932
Total Bilirubin, mg/dl	0.8 ± 0.3	0.4 ± 0.0	0.7 ± 0.1	1.2 ± 0.3	<0.001
Indirect Bilirubin, mg/dl	0.6 ± 0.2	0.3 ± 0.0	0.5 ± 0.1	0.9 ± 0.3	<0.001
Aspartate Transferase, IU/L	22 ± 5	21 ± 6	22 ± 5	22 ± 5	0.060
Alanine Transaminase, IU/L	20 ± 7	20 ± 7	20 ± 7	20 ± 7	0.668
Gamma-Glutamyl Transpeptidase, IU/L	122 ± 70	118 ± 79	119 ± 66	132 ± 77	0.063
Low-Density Lipoprotein Cholesterol, mg/dl	119 ± 28	116 ± 30	120 ± 28	117 ± 29	0.311
High-Density Lipoprotein Cholesterol, mg/dl	60 ± 16	53 ± 14	61 ± 16	61 ± 16	<0.001
Triglyceride, mg/dl	106 ± 65	132 ± 110	105 ± 62	99 ± 50	0.325
Hemoglobin A1c, %	5.8 ± 0.7	5.9 ± 0.7	5.8 ± 0.7	5.7 ± 0.7	0.174

The subjects were divided into three groups according to total bilirubin levels as follows: Low, <0.5 mg/dl; Intermediate, ≥0.5 mg/dl and <1.0 mg/dl; and High, ≥1.0 mg/dl.

### The Laboratory Data

The laboratory data are shown in Table 1. There were no significant differences in liver and biliary function tests among the groups. White blood cell count was higher and high-density lipoprotein (HDL) cholesterol was lower in the low-bilirubin group, compared with intermediate- and high- bilirubin groups. The levels of uric acid, an antioxidant product, were similar among the groups.

### The Prevalence of Severe DWMLs and the Related Variables

**Figure 1:**
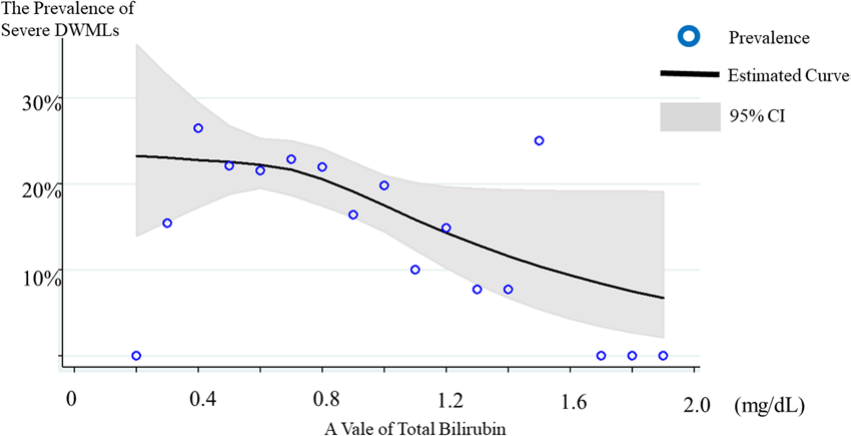
The association between total bilirubin levels and severe DWMLs. The cubic spline curve revealed an inverse association between bilirubin levels and severe DWMLs. DWML: deep white matter lesions.

The overall prevalence of severe DWMLs was 20.3%. Of these, the prevalence of the low-, intermediate-, and high-bilirubin groups was 24.1%, 21.3%, and 15.0%, respectively. Figure 1 shows the relationship between the prevalence of severe DWMLs and the total bilirubin levels, wherein a higher prevalence of severe DWMLs was observed in those with lower total bilirubin levels. Univariate logistic regression analysis revealed that age, hemoglobin A1c (HbA1c), systolic blood pressure (SBP), and past smoker were the factors associated with a higher prevalence of severe DWMLs, whereas alcohol consumption was related to a lower prevalence (Table 2). The prevalence of severe DWMLs increased as bilirubin levels decreased (*p* for trend = 0.026). The intermediate- and low-bilirubin groups exhibited a 1.53 (95% confidence interval (CI) = 1.02–2.29)- and 1.80 (95% CI = 0.96–3.36)-fold increase in the prevalence of severe DWMLs in comparison to the high-bilirubin group. After adjustment for multi-variables, the intermediate- and low-bilirubin groups exhibited a 1.33 (95% CI = 0.85–2.07)- and 2.36 (95% CI = 1.12–4.97)-fold increase in the prevalence of severe DWMLS, although the statistical significance of the linear trend test was attenuated (*p* for trend = 0.058).

**Table 2:** The Variables Related to Severe Deep White Matter Lesions.

	Univariate Logistic Analysis		Multivariate Logistic Analysis	
	Odds ratio (95% CI)	*p* value	Odds ratio (95% CI)	*p* value
Bilirubin Group				
High-Bilirubin Group	1.00	reference	1.00	reference
Intermediate-Bilirubin Group	1.53 (1.02–2.29)	0.040	1.33 (0.85–2.07)	0.214
Low-Bilirubin Group	1.80 (0.96–3.36)	0.065	2.36 (1.12–4.97)	0.024
		*p*_*for trend*_ = 0.026		*P*_*for trend*_ = 0.058
Age	1.12 (1.10–1.14)	<0.001	NA	NA
Male	1.08 (0.79–1.47)	0.644	NA	NA
Body Mass Index (1.0 increase)	1.03 (0.98–1.08)	0.230	NA	NA
Hemoglobin A1c (1% absolute increase)	1.39 (1.15–1.67)	0.001	NA	NA
Systolic Blood Pressure (10 mmHg increase)	1.22 (1.13–1.32)	<0.001	NA	NA
Smoke (vs never smoker)				
past smoker	1.44 (1.04–2.00)	0.027	NA	NA
current smoker	0.95 (0.59–1.52)	0.822	NA	NA
Alcohol (vs none)				
1–5 days a week	0.50 (0.35–0.72)	<0.001	NA	NA
≥5 days a week	0.68 (0.47–1.00)	0.048	NA	NA
Log-transformed White Blood Cell Count	1.51 (0.88–2.61)	0.135	NA	NA
Hemoglobin	0.97 (0.86–1.09)	0.587	NA	NA
High-Density Lipoprotein Cholesterol	1.00 (1.00–1.01)	0.730	NA	NA
Low-Density Lipoprotein Cholesterol	1.00 (1.00–1.01)	0.835	NA	NA
Albumin	0.89 (0.50–1.59)	0.697	NA	NA
Uric Acid	1.09 (0.97–1.22)	0.135	NA	NA

Multivariate logistic analysis was adjusted for age, gender, body mass index, hemoglobin A1c, systolic blood pressure, smoking, alcohol consumption, log-transformed white blood cell count, hemoglobin, high-density lipoprotein cholesterol, low-density lipoprotein cholesterol, albumin, and uric acid.

NA: Not Applicable.

### The Prevalence of PVH (periventricular hyperintensities) and the Related Variables

Severe PVH was observed in 84 subjects (7.5%) and not related to bilirubin in the uni- and multivariate logistic regression analysis.

### Interrater Reliability Regarding Rating of DWMLs and PVH

To evaluate interrater reliability of rating, 150 subjects were randomly selected. Weighted kappa statistics indicated almost perfect agreement (DWMLs: kappa = 0.87 ± 0.06; PVH: kappa = 0.80 ± 0.05, respectively).

## Discussion

Previous studies have demonstrated an inverse relationship between serum bilirubin levels and the prevalence of cardiovascular diseases^11–15^. The present study is the first report that reveals low total bilirubin levels could be associated with a high prevalence of severe DWMLs, which are related to impaired cognitive function, in apparently healthy subjects. Although bilirubin has antioxidant and anti-inflammatory properties and may suppress the development of atherosclerosis *in vitro*^9,15^, these observational studies are not sufficient to determine whether bilirubin would play such roles in the clinical setting as well.

A previous study, which analyzed the association of bilirubin with atherosclerotic risk factors using instrumental variable (IV) method, demonstrated that bilirubin levels were inversely associated with cardiovascular risk factors including body mass index (BMI), HDL cholesterol, and total cholesterol^7^. The findings suggested that bilirubin might be a confounding factor. Another study which assessed the causal effect of bilirubin levels on stroke risk using the IV method showed no causal relationship^8^. However, recently, a basic research was conducted to determine whether bilirubin could prevent atherosclerosis^16^. Vogel ME, *et al*. elucidated that the administration of bilirubin to low-density lipoprotein receptor-deficient mice impeded plaque formation and significantly reduced the infiltration of monocytes and lymphocytes into aortic root lesions by inhibiting endothelial vascular cell adhesion molecule 1 (VCAM-1) and intercellular adhesion molecule 1 (ICAM-1). Furthermore, they demonstrated that bilirubin blocked VCAM-1- and ICAM-1-mediated migration of monocytes across activated endothelial monolayers^16^. In the present study, as the inverse relationship was strengthened after adjustment for many possible confounding factors, the protective effect of bilirubin on DWMLs is considered a possibility although its efficacy may differ according to each vessel lesion. Currently, the findings concerning the causal effect of bilirubin are conflicting; thus, further studies are needed.

Serum bilirubin level is measurable in daily clinical practice around the world. We usually evaluate it in case of liver diseases or a suspicion of liver diseases. However, our study suggests that serum bilirubin level is an excellent potential marker for estimating the risk of severe DWMLs, which could be related to the development of stroke and dementia.

As the present study is a cross-sectional study, the causal relationship between bilirubin levels and DWMLs is unknown. However, as mentioned earlier, evaluation of bilirubin may be useful for an early documentation of the risk of dementia. The reason why the relationship between severe PVH and bilirubin was not observed may be due to insufficient number of the subjects with severe PVH.

In conclusion, bilirubin levels <0.5 mg/dl may indicate a higher prevalence of severe DWMLs in apparently healthy subjects.

## Methods

### Study Design and Population

This cross-sectional study included 1356 Japanese adults aged 40 years or older who were apparently healthy and initiatively underwent a health checkup program focusing on atherosclerosis between 2007 and 2011 at Tokyo Saiseikai Central Hospital. Although the evaluation of cognitive ability was not performed in the health checkup, the presence of cognitive impairment among the participants was unlikely because of their active participation in the health checkup and the capability of completing a self-reported questionnaire. Of these, individuals with abnormal liver enzymes (defined as aspartate transferase > 40 IU/L, alanine transaminase > 40 IU/L, or gamma-glutamyl transpeptidase > 90 IU/L) were excluded because of the possibility of having liver disease. The study protocol conforms to the ethical guidelines of the 1975 Declaration of Helsinki in line with the Ethical Guidelines for Epidemiological Research by the Japanese government. The study was approved by the ethics committee of Saiseikai Central Hospital (approval number 345). According to the guidelines, the study satisfied the conditions to waive the requirement for informed consent to individual participants. Therefore, informed consent was waived, and the ethics committee approved the waiver.

### Image modalities and conditions

The health checkup was performed focusing on atherosclerosis. Magnetic resonance imaging was performed to evaluate cerebral atherosclerosis using a 1.5-T scanner (SIGNA HDxt 1.5 T; GE Healthcare Japan, Tokyo, Japan). Hyperintensities on T2-weighted and FLAIR images of deep white matter were defined as DWMLs, indicating small-vessel disease. Periventricular hyperintensities on T2-weighted and FLAIR images was defined as PVH. Severity of DWMLs was rated on a scale of 0–3 according to the Fazekas scale: 0 refers to no white matter lesion, 1 refers to the presence of focal lesions, 2 refers to the beginning of confluent lesions, and 3 refers to the presence of diffuse involvement of the entire white matter^17,18^. Severe DWMLs were defined as lesions with Fazekas grade 3, which is reported to be associated with cognitive impairment^19^. We evaluated PVH grade according to Fazekas classification: 0 refers to absent, 1 refers to caps or pencil-thin lining, 2 refers to smooth halo, 3 refers to irregular PVH extending into the deep white matter^18^. PVH Fazekas with grade 3 was defined as severe PVH^20^. All ratings were performed by physicians under the direction of board certificated radiologist blind to the clinical data.

### The diagnosis of risk factors for atherosclerosis

Diabetes mellitus was diagnosed on the basis of the following criteria: self-reporting diabetes or the use of anti-diabetic medications based on the questionnaire, fasting plasma glucose ≥ 126 mg/dl, or HbA1c ≥ 6.5%^21^. Dyslipidemia was diagnosed if one of the following criteria was fulfilled: low-density lipoprotein cholesterol (calculated as total cholesterol – HDL cholesterol – triglyceride/5) ≥ 140 mg/dl, HDL cholesterol < 40 mg/dl, or triglyceride ≥ 150 mg/dl. Hypertension was defined as a SBP ≥ 140 mmHg or a diastolic blood pressure ≥ 90 mmHg^22^. Hyperuricemia was defined as a high serum uric acid value ≥ 7.0 mg/dl.

### Classification of Total Bilirubin

Total bilirubin was classified by every 0.5 mg/dl as following: low, <0.5 mg/dl; intermediate, ≥0.5 mg/dl and <1.0 mg/dl; and high, ≥1.0 mg/dl. We also analyzed the same population using tertiles of total bilirubin: low, <0.7 mg/dl; intermediate, ≥0.7 mg/dl and <0.9 mg/dl; high, ≥0.9 mg/dl. However, the analysis with tertile categorization might lack clinical relevance since the cutoffs vary according to the distribution of total bilirubin levels. In the present study, we demonstrated the result with more clinically relevant cutoffs. The result obtained by the tertile categorization was demonstrated in eTable 1.

### Statistical Analysis

Numerical data are presented as mean ± standard deviation. Categorical variables are expressed as absolute numbers (percentages). Continuous variables were analyzed using the unpaired Student’s *t*-test. The Fisher’s exact test and the χ^2^ test were used to analyze categorical variables. The associations between patients’ characteristics and the presence of severe DWMLs were assessed using uni- and multivariate logistic regression analyses and expressed in terms of odds ratio (OR), 95% CI, and *P* value. Clinically relevant variables (even without statistically significant differences) and variables with a p value < 0.10 in the univariate logistic regression analyses were used in the multivariate logistic regression analysis to determine whether bilirubin is an independent predictor of severe DWMLs. Consequently, in the multivariate logistic regression analyses, OR was adjusted for age, gender, BMI, HbA1c, SBP, smoking (never, past, and current smoker), the frequency of alcohol consumption (never, 1–4 days a week, ≥5 days a week), a natural logarithm of white blood cell counts, hemoglobin, albumin, uric acid, HDL cholesterol, and low-density lipoprotein cholesterol. In the multiple logistic regression, we did not stratify the analysis by gender, because no significant effect modification on the relationship by gender was observed. In this process, models with and without the interaction term of gender and each bilirubin group were constructed and compared using a chi-squared log-likelihood ratio test (χ2 = 0.78, *p* = 0.68), which found no significant effect modification. The similar uni- and multivariate logistic regression analysis were conducted regarding the prevalence of PVH. Weighted kappa statistics was used to examine interrater reliability between two raters regarding rating of DWMLs and PVH. Statistical significance was set at *p* < 0.05. All statistical analyses were carried out using Stata software, version 14 (StataCorp, College Station, TX).

## Electronic supplementary material

 Supplementary information
