# Supplementary material for: Low Bilirubin Levels Indicate a High Risk of Cerebral Deep White Matter Lesions in Apparently Healthy Subjects
Source: Sci Rep. 2018 Apr 24;8:6473. doi: 10.1038/s41598-018-24917-8 (PMC5915409; doi:10.1038/s41598-018-24917-8)
Supplement: Supplementary file 1 — Supplementary information [file 41598_2018_24917_MOESM1_ESM.docx]

Low Bilirubin Levels Indicate a High Risk of Cerebral Deep White Matter Lesions in Apparently Healthy Subjects

Satoshi Higuchi, MD, PhD^a^; Yusuke Kabeya, MD, MPH, PhD^b,c^; Junko Uchida, MD^d^; Kiyoe Kato, MD^d^; Nobuhiro Tsukada, MD, PhD^d^

^a^Department of Cardiology, Kyorin University Hospital, Tokyo, Japan

^b^Division of General Internal Medicine, Department of Internal Medicine, Tokai University, Kanagawa, Japan

^c^Department of Home Care Medicine, Saiyu Clinic, Saitama, Japan

^d^Department of Internal Medicine, Tokyo Saiseikai Central Hospital, Tokyo, Japan

eTable 1. Bilirubin Groups Cllasified by Tertile

|  | Univariate Logistic Analysis | | Multivariate Logistic Analysis | |
| --- | --- | --- | --- | --- |
|  | OR (95% CI) | *p* value | OR (95% CI) | *p* value |
| Bilirubin Group |  |  |  |  |
| High bilirubin group | 1.00 | reference | 1.00 | reference |
| Intermediate bilirubin group | 1.35 (0.97 - 1.88) | 0.078 | 1.28 (0.88 - 1.86) | 0.190 |
| Low bilirubin group | 1.40 (0.95 - 2.06) | 0.090 | 1.60 (0.99 - 2.57) | 0.053 |
|  |  | *p* *_for trend_* = 0.045 |  | *p* *_for trend_* = 0.049 |
|  |  |  |  |  |
